# Supplementary material for: Self- and informant-reported personality traits and vaccination against COVID-19
Source: PLoS One. 2024 Mar 14;19(3):e0287413. doi: 10.1371/journal.pone.0287413 (PMC10939290; doi:10.1371/journal.pone.0287413)
Supplement: Supplementary file 1 [file pone.0287413.s001.odt]

S1 Table

Sample Characteristics: Vaccination Details

| Characteristic | 100NP (N = 56,575) | NEO-PI-3 (N = 3,168) |
| --- | --- | --- |
| Vaccination timing |  |  |
| Priority-vaccinated (before May 17, 2021) | 31,339 (55.39%) | 2,042 (64.46%) |
| Early (May 17–August 31, 2021) | 15,566 (27.51%) | 587 (18.53%) |
| Late (from September 1, 2021) | 2,387 (4.22%) | 89 (2.81%) |
| Stage of vaccination |  |  |
| Unvaccinated | 7,283 (12.87%) | 450 (14.20%) |
| Primary series started | 1,754 (3.10%) | 60 (1.89%) |
| Primary series completed | 14,956 (26.44%) | 672 (21.21%) |
| Booster dose received | 32,582 (57.59%) | 1,986 (62.69%) |
| Vaccine received |  |  |
| Comirnaty (Pfizer-BioNTech) | 41,261 (72.93%) | 0 (0.00%) |
| Jcovden (Janssen) | 2,547 (4.50%) | 2,351 (74.21%) |
| Nuvaxovid (Novavax) | 54 (0.10%) | 0 (0.00%) |
| Spikevax (Moderna) | 9,176 (16.22%) | 406 (12.82%) |
| Vaxzevria (AstraZeneca) | 12,759 (22.55%) | 735 (23.20%) |

Note. Sums of vaccine types exceed sample sizes because some participants had received more than one type of vaccine.
