# Supplementary material for: Self- and informant-reported personality traits and vaccination against COVID-19
Source: PLoS One. 2024 Mar 14;19(3):e0287413. doi: 10.1371/journal.pone.0287413 (PMC10939290; doi:10.1371/journal.pone.0287413)
Supplement: Supplementary file 2 [file pone.0287413.s002.odt]

S2 Table

Informants’ Characteristics

| Characteristic | 100NP (N = 15,244) | NEO-PI-3 (N = 2,849) |
| --- | --- | --- |
| Age: mean (SD) | 45.18 (13.32) | 51.20 (15.49) |
| Sex |  |  |
| Male | 4,916 (32.25%) | 852 (29.91%) |
| Female | 10,328 (67.75%) | 1,997 (70.09%) |
| Education |  |  |
| With higher education | 10,358 (64.77%) | 1,235 (43.35%) |
| Without higher education | 5,634 (35.23%) | 1,614 (56.65%) |
| Relation type |  |  |
| Spouse or partner | 8,742 (57.35%) | 1,314 (47.84%) |
| Friend | 2,250 (14.76%) | 454 (16.20%) |
| Child or grandchild | 1,961 (12.86%) | 194 (6.92%) |
| Sister or brother | 1,048 (6.87%) | 175 (6.24%) |
| Parent or grandparent | 929 (6.09%) | 14  (0.50%) |
| Other relative | 179 (1.17%) | 543 (19.37%) |
| Other acquaintance | 135 (0.89%) | 82 (2.93%) |
