# Supplementary material for: Self- and informant-reported personality traits and vaccination against COVID-19
Source: PLoS One. 2024 Mar 14;19(3):e0287413. doi: 10.1371/journal.pone.0287413 (PMC10939290; doi:10.1371/journal.pone.0287413)
Supplement: Supplementary file 3 [file pone.0287413.s003.odt]

S3 Table

Items Assigned to the Five Domains in the 100NP

| Domain | Item | Reverse 1 |
| --- | --- | --- |
| A | Hate to hear about the successes of others | 1 |
| A | Am happy to see others doing well for themselves |  |
| A | Would like to have more power than other people | 1 |
| A | Want to be in charge | 1 |
| A | Love dangerous situations | 1 |
| A | Like to stand out in a crowd | 1 |
| A | Try to out do others | 1 |
| A | Like to compete and do everything I can to win | 1 |
| A | Find it easy to manipulate others | 1 |
| A | Use flattery to get ahead | 1 |
| A | Use others to get what I want | 1 |
| A | Would like for other people to be afraid of me | 1 |
| A | Have a sharp tongue | 1 |
| A | Can’t be bothered with others needs | 1 |
| A | Am quick to judge others | 1 |
| A | Get back at people who insult me | 1 |
| A | Believe that I am better than others | 1 |
| A | Insist on getting my way | 1 |
| A | Believe that I am always right | 1 |
| A | Blame others when something goes wrong | 1 |
| A | Can’t stand weak people | 1 |
| A | Am likely to show off if I get the chance | 1 |
| A | Admire a really clever scam | 1 |
| A | Enjoy hurting others | 1 |
| A | Know how to get around the rules | 1 |
| A | Cheat to get ahead | 1 |
| A | Am mainly interested in money | 1 |
| A | Try to provoke others just to get a response | 1 |
| C | Think good manners are very important |  |
| C | Like to be viewed as proper and conventional |  |
| C | Love order and regularity |  |
| C | Am always prepared |  |
| C | Do things in a logical order |  |
| C | Am always on time |  |
| C | Hardly ever finish things on time | 1 |
| C | Work hard |  |
| C | Keep things tidy |  |
| C | Leave a mess in my room | 1 |
| C | Break my promises | 1 |
| C | Keep my promises |  |
| C | Start tasks right away |  |
| C | Don’t quit a task before it is finished |  |
| C | Continue until everything is perfect |  |
| C | Want every detail taken care of |  |
| C | Avoid responsibilities | 1 |
| C | Get upset if objects are not arranged properly |  |
| E | Prefer to be alone | 1 |
| E | Talk a lot |  |
| E | Enjoy social gatherings |  |
| E | Talking with others energizes me |  |
| E | Have no need for close friendships | 1 |
| E | Having good friends is important for me |  |
| E | Make friends easily |  |
| E | Find that nothing excites me | 1 |
| E | Have a lot of fun |  |
| E | Smile a lot |  |
| E | Experience my emotions intensely |  |
| E | Am open about my feelings |  |
| E | Have difficulty showing affection | 1 |
| E | Dislike talking about myself | 1 |
| E | Enjoy cooperating with others |  |
| E | Like being part of a team |  |
| E | Love to look my best |  |
| N | Am always worried about something |  |
| N | Am afraid of many things |  |
| N | Rarely worry | 1 |
| N | Feel restless a lot of the time |  |
| N | Often feel blue |  |
| N | Have frequent mood swings |  |
| N | Am not embarrassed easily | 1 |
| N | Worry about what people think of me |  |
| N | Get stressed out easily |  |
| N | Often feel lonely |  |
| N | Have a dark outlook on the future |  |
| N | Tend to feel very hopeless |  |
| N | Become anxious in new situations |  |
| N | Have a low opinion of myself |  |
| N | Feel that my life lacks direction |  |
| N | Am happy with my life | 1 |
| N | Often feel that others misunderstand me |  |
| N | Cry easily |  |
| N | My feelings are easily hurt |  |
| N | Need reassurance |  |
| N | Am afraid of being left alone |  |
| N | Am often troubled by feelings of guilt |  |
| N | Let others take advantage of me |  |
| N | Am a submissive person |  |
| N | Often feel tired |  |
| N | Am not easily affected by my emotions | 1 |
| N | Often feel that others laugh or talk about me |  |
| N | Can’t make up my mind |  |
| N | Postpone decisions |  |
| N | Often need help |  |
| N | Spend a lot of time thinking about past mistakes |  |
| O | Take risks |  |
| O | Am considered to be kind of eccentric |  |
| O | Avoid philosophical discussions | 1 |
| O | Love to learn new things |  |
| O | Am interested in science |  |
| O | Believe in the importance of art |  |
| O | Love to think up new ways of doing things |  |
| O | Need a creative outlet |  |
| O | Have a rich vocabulary |  |
| O | Like to solve complex problems |  |
| O | Have a vivid imagination |  |
| O | Am interested in many things |  |

1 Items marked with 1 are reverse scored.
