# Supplementary material for: Self- and informant-reported personality traits and vaccination against COVID-19
Source: PLoS One. 2024 Mar 14;19(3):e0287413. doi: 10.1371/journal.pone.0287413 (PMC10939290; doi:10.1371/journal.pone.0287413)
Supplement: Supplementary file 4 [file pone.0287413.s004.odt]

S4 Table

Predicting Vaccination from Personality Traits

| Predictors | Source: self | Source: informant |
| --- | --- | --- |
| r [95% CI] a | Error rate [95% CI] | p b | n (training) | n (testing) | r [95% CI] a | Error rate [95% CI] | p b | n (training) | n (testing) |
| DV: binary vaccination status |
| Domains | .06 [.03; .10] | .47 [.45; .48] | < .001 | 10,924 | 3,642 | .08 [.02; .15] | .46 [.43; .50] | .031 | 2,479 | 827 |
| Items | .31 [.28; .34] | .34 [.33; .36] | < .001 | 10,924 | 3,642 | .24 [.18; .31] | .38 [.35; .41] | < .001 | 2,479 | 827 |
| DV: stage of vaccination |
| Domains | .05 [.03; .06] | – | < .001 | 42,431 | 14,144 | .02 [-.01; .06] | – | .134 | 11,433 | 3,811 |
| Items | .31 [.29; .32] | – | < .001 | 42,431 | 14,144 | .24 [.21; .27] | – | < .001 | 11,433 | 3,811 |
| DV: vaccination timing |
| Domains | .00 [-.05; .06] | .50 [.48; .53] | .795 | 3,580 | 1,194 | .08 [-.04; .20] | .46 [.40; .52] | .250 | 837 | 279 |
| Items | .18 [.13; .24] | .41 [.38; .44] | < .001 | 3,580 | 1,194 | .14 [.02; .25] | .43 [.37; .49] | .045 | 837 | 279 |

Note. All predictors were residualized for age, sex, and education. Out-of-sample predictions (i.e., associations in the testing samples) are reported.

a Pearson’s correlation between predicted and actual number of doses (stage of vaccination) or Matthew’s correlation, calculated based on the binary outcomes’ predicted and actual values.

b The p-value associated with the correlation (stage of vaccination) or error rate (binary outcomes), corrected using FDR.
