# Supplementary material for: Self- and informant-reported personality traits and vaccination against COVID-19
Source: PLoS One. 2024 Mar 14;19(3):e0287413. doi: 10.1371/journal.pone.0287413 (PMC10939290; doi:10.1371/journal.pone.0287413)
Supplement: Supplementary file 5 [file pone.0287413.s005.odt]

S5 Table

Prediction of Vaccination from Earlier Personality Measurements With the NEO-PI-3

| Predictors | Source: self | Source: informant |
| --- | --- | --- |
| r [95% CI] a | Error rate [95% CI] | p b | n (training) | n (testing) | r [95% CI] a | Error rate [95% CI] | p b | n (training) | n (testing) |
| DV: binary vaccination status |
| Domains | .03 [-.10; .16] | .49 [.42; .56] | .892 | 675 | 225 | .01 [-.12; .15] | .49 [.42; .56] | .892 | 649 | 217 |
| Facets | .14 [.01; .27] | .43 [.37; .50] | .136 | 675 | 225 | .15 [.02; .28] | .42 [.36; .49] | .136 | 649 | 217 |
| Items | .05 [-.08; .18] | .48 [.41; .54] | .758 | 675 | 225 | .07 [-.06; .20] | .47 [.40; .53] | .684 | 649 | 217 |
| DV: stage of vaccination |
| Domains | – c | – | – | 2,376 | 792 | .00 [-.07; .07] | – | .946 | 2,260 | 754 |
| Facets | .05 [-.02; .12] | – | .420 | 2,376 | 792 | .03 [-.05; .10] | – | .609 | 2,260 | 754 |
| Items | .07 [.00; .14] | – | .200 | 2,376 | 792 | .04 [-.04; .11] | – | .546 | 2,260 | 754 |
| DV: vaccination timing |
| Domains | .26 [-.04; .51] | .38 [.24; .53] | .405 | 133 | 45 | .09 [-.21; .38] | .45 [.30; .61] | .766 | 130 | 44 |
| Facets | .29 [.00; .54] | .36 [.22; .51] | .405 | 133 | 45 | .18 [-.13; .45] | .41 [.26; .57] | .437 | 130 | 44 |
| Items | -.07 [-.35; .23] | .53 [.38; .68] | .766 | 133 | 45 | .18 [-.12; .46] | .41 [.26; .57] | .437 | 130 | 44 |

Note. All predictors (domains, facets, and items) were residualized for age, sex, and education. Out-of-sample predictions (i.e., associations in the testing samples) are reported.

a Pearson’s correlation between predicted and actual number of doses (stage of vaccination) or Matthew’s correlation, calculated based on the binary outcomes’ predicted and actual values.

b The p-value associated with the correlation (stage of vaccination) or error rate (binary outcomes), corrected using FDR.

c The domains were unable to predict stage of vaccination (elastic net weights were zero for all predictors).
