# Supplementary material for: Self- and informant-reported personality traits and vaccination against COVID-19
Source: PLoS One. 2024 Mar 14;19(3):e0287413. doi: 10.1371/journal.pone.0287413 (PMC10939290; doi:10.1371/journal.pone.0287413)
Supplement: Supplementary file 6 [file pone.0287413.s006.odt]

S6 Table

Prediction Accuracies of Personality Items and Demographic Variables

| Model | Error rate [95% CI] | r [95% CI] a |
| --- | --- | --- |
| Items | .29 [.27, .31] | .42 [.39, .45] |
| Demographics | .38 [.36, .39] | .25 [.22, .28] |
| Items + demographics | .28 [.27, .30] | .43 [.40, .46] |

Note. N = 12,014 (9,010 used for training and 3,004 for testing the models). The demographic variables were age, sex, education, occupation, and residency.

a Matthew’s correlation, calculated based on the binary outcomes’ predicted and actual values.
