# Supplementary material for: Self- and informant-reported personality traits and vaccination against COVID-19
Source: PLoS One. 2024 Mar 14;19(3):e0287413. doi: 10.1371/journal.pone.0287413 (PMC10939290; doi:10.1371/journal.pone.0287413)
Supplement: Supplementary file 7 [file pone.0287413.s007.odt]

S7 Table

Items’ Elastic Net Weights

| Item | Raw items | Residualized items |
| --- | --- | --- |
| Am always worried about something | 0.0288 | 0.0000 |
| Rarely worry | 0.0061 | 0.0000 |
| Am relaxed most of the time | -0.0251 | -0.0083 |
| Feel restless a lot of the time | 0.0398 | 0.0045 |
| Often feel blue | 0.0188 | 0.0000 |
| Try to avoid speaking in public | -0.1341 | -0.1069 |
| Am not embarrassed easily | 0.0636 | 0.0459 |
| Worry about what people think of me | 0.0620 | 0.0509 |
| Easily resist temptations | 0.0016 | 0.0000 |
| Am satisfied with my relationships | 0.0144 | 0.0000 |
| Have a dark outlook on the future | -0.0223 | 0.0000 |
| Tend to feel very hopeless | -0.0454 | -0.0006 |
| Become anxious in new situations | -0.0686 | -0.0138 |
| Feel that my life lacks direction | -0.0101 | 0.0000 |
| Am happy with my life | -0.0733 | -0.0380 |
| Often feel that others misunderstand me | -0.0301 | -0.0291 |
| Am happy to see others doing well for themselves | -0.0542 | -0.0371 |
| Worry about my health | 0.0258 | 0.0036 |
| Consider myself healthy for my age | -0.0504 | -0.0238 |
| Have been richly blessed in my life | 0.0645 | 0.0494 |
| Prefer to be alone | -0.0124 | 0.0000 |
| Talk a lot | 0.0013 | 0.0000 |
| Enjoy social gatherings | -0.0585 | 0.0000 |
| Talking with others energizes me | -0.0292 | 0.0000 |
| Have no need for close friendships | 0.0047 | 0.0000 |
| Act as a leader | 0.0374 | 0.0000 |
| Would like to have more power than other people | 0.0267 | 0.0000 |
| Let others take advantage of me | 0.0160 | 0.0000 |
| Am able to stand up for myself | -0.0415 | -0.0254 |
| Often feel tired | 0.0282 | 0.0000 |
| Take risks | -0.0255 | 0.0000 |
| Love excitement | -0.0096 | -0.0140 |
| Am always busy | 0.0528 | 0.0211 |
| Am often bored | 0.0048 | 0.0000 |
| Find that nothing excites me | 0.0140 | 0.0000 |
| Have a lot of fun | 0.0231 | 0.0000 |
| Smile a lot | -0.0135 | 0.0000 |
| Am always joking | -0.0171 | 0.0000 |
| Think good manners are very important | -0.0145 | 0.0000 |
| Support liberal political candidates | 0.3382 | 0.3042 |
| Am considered to be kind of eccentric | -0.1701 | -0.1649 |
| Rebel against authority | -0.0831 | -0.0379 |
| Respect authority | 0.0255 | 0.0000 |
| Avoid philosophical discussions | -0.0804 | -0.0270 |
| Find political discussions interesting | 0.1267 | 0.1059 |
| Like to read | 0.1082 | 0.1054 |
| Love to learn new things | -0.0571 | -0.0175 |
| Am interested in science | 0.0936 | 0.0534 |
| Believe in the importance of art | 0.1906 | 0.1538 |
| Get deeply immersed in music | -0.0298 | -0.0098 |
| Love to think up new ways of doing things | -0.0123 | 0.0000 |
| Need a creative outlet | -0.0206 | 0.0000 |
| Have a rich vocabulary | 0.0518 | 0.0252 |
| Learn quickly | -0.0734 | -0.0521 |
| Dislike routine | -0.0622 | -0.0359 |
| Like to visit new places | 0.0504 | 0.0000 |
| Have difficulty showing affection | -0.0076 | 0.0000 |
| Believe that everyone should have a say | -0.2216 | -0.1935 |
| Trust others | 0.0734 | 0.0511 |
| Get suspicious when someone treats me nicely | -0.0420 | -0.0242 |
| Often feel that others laugh or talk about me | -0.0357 | 0.0000 |
| Try to out do others | -0.0464 | 0.0000 |
| Have a natural talent for influencing people | 0.0268 | 0.0000 |
| Use flattery to get ahead | -0.0216 | 0.0000 |
| Think of others first | -0.0025 | 0.0000 |
| Make enemies | 0.0787 | 0.0435 |
| Have a sharp tongue | -0.0248 | 0.0000 |
| Yell at people | -0.0603 | -0.0137 |
| Don’t hesitate to express an unpopular opinion | -0.0681 | -0.0489 |
| Can’t stand confrontations | -0.0060 | 0.0000 |
| Love to help others | -0.0268 | -0.0045 |
| Am good at understanding others feelings | -0.0384 | -0.0089 |
| Am quick to correct others | -0.0281 | 0.0000 |
| Am quick to judge others | -0.0614 | -0.0326 |
| Get back at people who insult me | -0.0183 | 0.0000 |
| Believe that I am always right | 0.0443 | 0.0007 |
| Believe that we should be tough on crime | -0.0273 | -0.0056 |
| Can’t stand weak people | -0.0281 | 0.0000 |
| Believe that the poor deserve our sympathy | 0.0596 | 0.0199 |
| Admire a really clever scam | 0.0847 | 0.0549 |
| Enjoy cooperating with others | 0.0500 | 0.0057 |
| Like being part of a team | 0.1103 | 0.0749 |
| Enjoy hurting others | -0.0328 | -0.0012 |
| Am always prepared | 0.0135 | 0.0000 |
| Do things in a logical order | -0.0010 | 0.0000 |
| Am always on time | 0.0405 | 0.0088 |
| Hardly ever finish things on time | 0.0175 | 0.0000 |
| Leave a mess in my room | -0.0017 | 0.0000 |
| Start tasks right away | -0.0207 | 0.0000 |
| Act without thinking | 0.0179 | 0.0000 |
| Am considered to be a wise person | 0.0115 | 0.0000 |
| Can’t make up my mind | -0.0195 | 0.0000 |
| Postpone decisions | -0.0108 | 0.0000 |
| Don’t quit a task before it is finished | 0.0033 | 0.0000 |
| Often stop working if it becomes too hard | 0.0186 | 0.0000 |
| Am good at many things | -0.0426 | -0.0228 |
| Want to get things done without anyones help | -0.0078 | -0.0053 |
| Am easily distracted | 0.0358 | 0.0000 |
| Often forget things | -0.0742 | -0.0348 |
| Continue until everything is perfect | -0.0340 | -0.0181 |
| Want every detail taken care of | -0.0575 | -0.0415 |
| Know how to get around the rules | 0.0631 | 0.0388 |
| Cheat to get ahead | 0.0047 | 0.0000 |
| Often tell lies | 0.0303 | 0.0029 |
| Am an extremely loyal person | -0.0347 | -0.0184 |
| Believe one has special duties to ones family | -0.0150 | 0.0000 |
| Spend more money than I should | 0.0041 | 0.0000 |
| Love luxury | -0.0547 | -0.0016 |
| Am mainly interested in money | -0.0088 | 0.0000 |
| Enjoy flirting | -0.0185 | 0.0000 |
| Am a spiritual person | -0.3678 | -0.3454 |
| Believe that all events can be explained scientifically | 0.3729 | 0.3473 |
| Wear stylish clothing | 0.0318 | 0.0000 |
| Love to look my best | 0.0264 | 0.0000 |
| Consider myself good-looking | -0.1038 | -0.0770 |
| Believe in the power of fate | -0.1569 | -0.1434 |
| Get upset if objects are not arranged properly | -0.0280 | -0.0055 |
| Spend a lot of time thinking about past mistakes | 0.1176 | 0.0788 |
| Easily laugh at myself | 0.0021 | 0.0000 |
| Experience deep emotions when I see beautiful things | 0.0158 | 0.0000 |
| Work on improving myself | -0.0123 | 0.0000 |
| Consider myself a traditionally masculine person | -0.0066 | 0.0000 |

Note. Self-reported items’ weights in elastic net models predicting binary vaccination status are shown. For residualized weights, the items were residualized for for age, sex, and education before running the model, which was trained on 10,924 people and tested on 3,642 people. The raw (unresidualized) items’ weights were obtained from the model trained on 9,010 people and tested on 3,004 people (people with full demographic data). Items with weights equal to zero in both models are not displayed.
