# Supplementary material for: Self- and informant-reported personality traits and vaccination against COVID-19
Source: PLoS One. 2024 Mar 14;19(3):e0287413. doi: 10.1371/journal.pone.0287413 (PMC10939290; doi:10.1371/journal.pone.0287413)
Supplement: Supplementary file 8 [file pone.0287413.s008.odt]

S8 Table

Demographic Variables as Moderators in the Associations Between Personality Traits and Vaccination Status

| Source | Predictors | Error rate (training) | Error rate (testing) | Difference [95% CI] | χ2 a | p | n (training) | n (testing) |
| --- | --- | --- | --- | --- | --- | --- | --- | --- |
| Moderator: age |
| Self | Domains | .44 | .59 | -.15 [-.17; -.13] | 243.22 | < .001 | 5,546 | 5,546 |
| Self | Items | .35 | .40 | -.05 [-.07; -.04] | 34.40 | < .001 | 5,546 | 5,546 |
| Informant | Domains | .46 | .58 | -.12 [-.16; -.07] | 29.03 | < .001 | 1,061 | 1,061 |
| Informant | Items | .39 | .38 | .02 [-.03; .06] | 0.51 | .475 | 1,061 | 1,061 |
| Moderator: sex |
| Self | Domains | .42 | .49 | -.07 [-.09; -.05] | 39.88 | < .001 | 3,935 | 3,935 |
| Self | Items | .34 | .38 | -.04 [-.06; -.01] | 10.50 | .002 | 3,935 | 3,935 |
| Informant | Domains | .41 | .52 | -.11 [-.15; -.06] | 22.11 | < .001 | 945 | 945 |
| Informant | Items | .37 | .42 | -.05 [-.10; -.01] | 5.10 | .024 | 945 | 945 |
| Moderator: education |
| Self | Domains | .41 | .57 | -.15 [-.17; -.14] | 331.28 | < .001 | 7,031 | 7,031 |
| Self | Items | .31 | .44 | -.13 [-.15; -.11] | 252.28 | < .001 | 7,031 | 7,031 |
| Informant | Domains | .43 | .61 | -.18 [-.22; -.14] | 80.13 | < .001 | 1,265 | 1,265 |
| Informant | Items | .35 | .46 | -.11 [-.15; -.07] | 32.60 | < .001 | 1,265 | 1,265 |
| Moderator: residency |
| Self | Domains | .47 | .49 | -.02 [-.04; .00] | 2.52 | .224 | 3,672 | 3,672 |
| Self | Items | .34 | .36 | -.02 [-.04; .00] | 2.53 | .224 | 3,672 | 3,672 |
| Informant | Domains | .51 | .53 | -.01 [-.06; .04] | 0.20 | .655 | 813 | 813 |
| Informant | Items | .35 | .37 | -.02 [-.07; .03] | 0.52 | .625 | 813 | 813 |
| Moderator: occupation |
| Self | Domains | .46 | .54 | -.08 [-.10; -.05] | 31.75 | < .001 | 2,778 | 2,778 |
| Self | Items | .35 | .45 | -.10 [-.12; -.07] | 54.55 | < .001 | 2,778 | 2,778 |
| Informant | Domains | .44 | .69 | -.25 [-.31; -.19] | 59.64 | < .001 | 475 | 475 |
| Informant | Items | .41 | .49 | -.08 [-.15; -.01] | 5.82 | .016 | 475 | 475 |

Note. For the moderators age, sex, education, residency, and occupation, testing data consisted of either participants who were younger, male, had higer education, lived in urban areas, or had white-collar occupations, respectively; training data consisted of participants who were older, female, had no higher education, lived in rural areas, or held blue-collar occupations, respectively.

a df = 1.
