# Supplementary material for: Self- and informant-reported personality traits and vaccination against COVID-19
Source: PLoS One. 2024 Mar 14;19(3):e0287413. doi: 10.1371/journal.pone.0287413 (PMC10939290; doi:10.1371/journal.pone.0287413)
Supplement: Supplementary file 9 [file pone.0287413.s009.odt]

S9 Table

Big Five Domains’ Associations With Vaccination Status (100NP)

| Domain | Source: self | Source: informant |
| --- | --- | --- |
| OR [95% CI] | p | OR [95% CI] | p |
| All participants |
| Neuroticism | 1.10 [1.07; 1.13] | < .001 | 1.02 [0.97; 1.08] | .612 |
| Extraversion | 1.03 [1.00; 1.05] | .081 | 1.02 [0.97; 1.07] | .629 |
| Openness | 0.88 [0.86; 0.91] | < .001 | 0.91 [0.87; 0.96] | .005 |
| Agreeableness | 1.00 [0.97; 1.03] | .941 | 1.07 [1.02; 1.13] | .017 |
| Conscientiousness | 0.99 [0.96; 1.01] | .344 | 1.01 [0.96; 1.07] | .641 |
| Without higher education |
| Neuroticism | 1.08 [1.04; 1.12] | < .001 | 1.06 [0.98; 1.15] | .262 |
| Extraversion | 1.02 [0.98; 1.05] | .313 | 1.07 [0.99; 1.16] | .233 |
| Openness | 0.91 [0.88; 0.95] | < .001 | 0.97 [0.89; 1.05] | .513 |
| Agreeableness | 1.03 [0.99; 1.07] | .129 | 1.08 [1.00; 1.16] | .233 |
| Conscientiousness | 0.97 [0.94; 1.00] | .116 | 0.98 [0.91; 1.06] | .685 |
| Blue-collar employment |
| Neuroticism | 1.08 [1.02; 1.14] | .012 | 1.02 [0.90; 1.15] | .824 |
| Extraversion | 1.01 [0.95; 1.06] | .794 | 1.10 [0.97; 1.24] | .321 |
| Openness | 0.87 [0.83; 0.92] | < .001 | 0.99 [0.87; 1.11] | .824 |
| Agreeableness | 1.06 [1.00; 1.12] | .073 | 1.16 [1.03; 1.30] | .084 |
| Conscientiousness | 0.97 [0.92; 1.02] | .284 | 0.98 [0.87; 1.10] | .824 |
