# Supplementary material for: Self- and informant-reported personality traits and vaccination against COVID-19
Source: PLoS One. 2024 Mar 14;19(3):e0287413. doi: 10.1371/journal.pone.0287413 (PMC10939290; doi:10.1371/journal.pone.0287413)
Supplement: Supplementary file 10 [file pone.0287413.s010.odt]

S10 Table

Personality Items Most Strongly Associated With Vaccination Status

| Nr | Item | Source: self | Source: informant |
| --- | --- | --- | --- |
| OR [95% CI] | p | OR [95% CI] | p |
| #1 | Believe that all events can be explained scientifically | 1.60 [1.56; 1.64] | < .001 | 1.50 [1.42; 1.58] | < .001 |
| #2 | Am a spiritual person | 0.63 [0.62; 0.65] | < .001 | 0.66 [0.63; 0.70] | < .001 |
| #3 | Support liberal political candidates | 1.36 [1.33; 1.40] | < .001 | 1.37 [1.30; 1.44] | < .001 |
| #4 | Believe in the power of fate | 0.74 [0.72; 0.76] | < .001 | 0.68 [0.65; 0.72] | < .001 |
| #5 | Worry about what people think of me | 1.21 [1.18; 1.24] | < .001 | 1.12 [1.07; 1.19] | < .001 |
| #6 | Am considered to be kind of eccentric | 0.80 [0.78; 0.82] | < .001 | 0.84 [0.79; 0.88] | < .001 |
| #7 | Don’t consider myself religious | 1.19 [1.16; 1.22] | < .001 | 1.16 [1.11; 1.22] | < .001 |
| #8 | Rebel against authority | 0.84 [0.82; 0.87] | < .001 | 0.78 [0.74; 0.82] | < .001 |
| #9 | Believe that everyone should have a say | 0.85 [0.83; 0.87] | < .001 | – | – |
| #10 | Respect authority | 1.14 [1.12; 1.17] | < .001 | 1.16 [1.11; 1.23] | < .001 |
| #11 | Know how to get around the rules | 0.86 [0.83; 0.88] | < .001 | 0.86 [0.81; 0.90] | < .001 |
| #12 | Like being part of a team | 1.14 [1.12; 1.17] | < .001 | – | – |
| #13 | Am good at many things | 0.87 [0.85; 0.89] | < .001 | – | – |
| #14 | Like to be viewed as proper and conventional | 1.13 [1.10; 1.16] | < .001 | – | – |
| #15 | Consider myself good-looking | 0.87 [0.85; 0.90] | < .001 | 0.89 [0.84; 0.93] | < .001 |
| #16 | Am always worried about something | 1.12 [1.10; 1.15] | < .001 | – | – |
| #17 | Am interested in science | 1.12 [1.09; 1.15] | < .001 | 1.18 [1.12; 1.25] | < .001 |
| #18 | Need reassurance | 1.12 [1.09; 1.15] | < .001 | – | – |
| #19 | Take risks | 0.88 [0.86; 0.90] | < .001 | 0.87 [0.82; 0.92] | < .001 |
| #20 | Dislike routine | 0.88 [0.86; 0.90] | < .001 | – | – |
| #21 | Treat all ethnicities and religions equally | – | – | 1.15 [1.09; 1.21] | < .001 |
| #22 | Do things that women traditionally do | – | – | 0.86 [0.81; 0.92] | < .001 |
| #23 | Need a creative outlet | – | – | 0.87 [0.82; 0.92] | < .001 |
| #24 | Often feel that others misunderstand me | – | – | 0.88 [0.84; 0.93] | < .001 |
| #25 | Love to daydream | – | – | 0.88 [0.84; 0.93] | < .001 |
| #26 | Don’t hesitate to express an unpopular opinion | – | – | 0.88 [0.84; 0.93] | < .001 |
| #27 | Adapt easily to new situations | – | – | 0.88 [0.84; 0.93] | < .001 |

Note. Positive odds ratios indicate that higher values of the trait are associated with higher odds of being vaccinated; negative odds ratios indicate that higher values of the trait are associated with lower odds of being vaccinated.
