# Supplementary material for: Self- and informant-reported personality traits and vaccination against COVID-19
Source: PLoS One. 2024 Mar 14;19(3):e0287413. doi: 10.1371/journal.pone.0287413 (PMC10939290; doi:10.1371/journal.pone.0287413)
Supplement: Supplementary file 11 [file pone.0287413.s011.odt]

S11 Table

Personality Items Most Strongly Associated With Vaccination Status Among People Without Higher Education

| Nr | Item | Source: self | Source: informant |
| --- | --- | --- | --- |
| OR [95% CI] | p | OR [95% CI] | p |
| #1 | Believe that all events can be explained scientifically | 1.49 [1.44; 1.54] | < .001 | 1.44 [1.33; 1.56] | < .001 |
| #2 | Support liberal political candidates | 1.37 [1.33; 1.42] | < .001 | 1.36 [1.27; 1.47] | < .001 |
| #3 | Am a spiritual person | 0.67 [0.65; 0.70] | < .001 | 0.68 [0.63; 0.74] | < .001 |
| #4 | Believe in the power of fate | 0.74 [0.71; 0.76] | < .001 | 0.74 [0.68; 0.80] | < .001 |
| #5 | Am considered to be kind of eccentric | 0.80 [0.77; 0.82] | < .001 | 0.83 [0.77; 0.90] | < .001 |
| #6 | Worry about what people think of me | 1.19 [1.15; 1.23] | < .001 | 1.14 [1.05; 1.23] | .002 |
| #7 | Don’t consider myself religious | 1.18 [1.14; 1.22] | < .001 | 1.15 [1.06; 1.24] | .001 |
| #8 | Rebel against authority | 0.86 [0.83; 0.89] | < .001 | 0.79 [0.73; 0.85] | < .001 |
| #9 | Like being part of a team | 1.14 [1.10; 1.18] | < .001 | – | – |
| #10 | Dislike routine | 0.87 [0.84; 0.90] | < .001 | – | – |
| #11 | Am able to stand up for myself | 0.87 [0.84; 0.90] | < .001 | – | – |
| #12 | Believe that everyone should have a say | 0.87 [0.84; 0.90] | < .001 | – | – |
| #13 | Don’t hesitate to express an unpopular opinion | 0.87 [0.84; 0.90] | < .001 | 0.84 [0.78; 0.91] | < .001 |
| #14 | Know how to get around the rules | 0.87 [0.84; 0.90] | < .001 | 0.86 [0.79; 0.93] | < .001 |
| #15 | Am interested in science | 1.12 [1.09; 1.16] | < .001 | 1.20 [1.11; 1.30] | < .001 |
| #16 | Love to think up new ways of doing things | 0.88 [0.85; 0.91] | < .001 | – | – |
| #17 | Trust others | 1.12 [1.08; 1.16] | < .001 | – | – |
| #18 | Like to read | 1.11 [1.08; 1.15] | < .001 | 1.15 [1.07; 1.24] | < .001 |
| #19 | Am afraid of being left alone | 1.11 [1.07; 1.15] | < .001 | – | – |
| #20 | Am a submissive person | 1.11 [1.07; 1.15] | < .001 | – | – |
| #21 | Respect authority | – | – | 1.22 [1.13; 1.32] | < .001 |
| #22 | Am not embarrassed easily | – | – | 0.82 [0.76; 0.89] | < .001 |
| #23 | Find political discussions interesting | – | – | 1.17 [1.07; 1.27] | < .001 |
| #24 | Love dangerous situations | – | – | 0.84 [0.78; 0.91] | < .001 |
| #25 | Worry a lot about my looks | – | – | 1.14 [1.06; 1.24] | .001 |
| #26 | Enjoy cooperating with others | – | – | 1.14 [1.06; 1.23] | .001 |
| #27 | Easily apologize when I have been wrong | – | – | 1.13 [1.05; 1.23] | .002 |
| #28 | Do things that women traditionally do | – | – | 0.87 [0.79; 0.96] | .004 |
