# Supplementary material for: Self- and informant-reported personality traits and vaccination against COVID-19
Source: PLoS One. 2024 Mar 14;19(3):e0287413. doi: 10.1371/journal.pone.0287413 (PMC10939290; doi:10.1371/journal.pone.0287413)
Supplement: Supplementary file 12 [file pone.0287413.s012.odt]

S12 Table

Personality Items Most Strongly Associated With Vaccination Status Among People in Blue-Collar Occupations

| Nr | Item | Source: self | Source: informant |
| --- | --- | --- | --- |
| OR [95% CI] | p | OR [95% CI] | p |
| #1 | Believe that all events can be explained scientifically | 1.34 [1.27; 1.41] | < .001 | 1.41 [1.24; 1.60] | < .001 |
| #2 | Am a spiritual person | 0.68 [0.64; 0.72] | < .001 | 0.66 [0.58; 0.75] | < .001 |
| #3 | Believe in the power of fate | 0.76 [0.71; 0.80] | < .001 | 0.79 [0.70; 0.90] | .001 |
| #4 | Support liberal political candidates | 1.24 [1.17; 1.30] | < .001 | 1.31 [1.18; 1.47] | < .001 |
| #5 | Worry about what people think of me | 1.19 [1.13; 1.26] | < .001 | – | – |
| #6 | Am considered to be kind of eccentric | 0.82 [0.78; 0.87] | < .001 | – | – |
| #7 | Know how to get around the rules | 0.84 [0.80; 0.89] | < .001 | – | – |
| #8 | Dislike routine | 0.85 [0.81; 0.90] | < .001 | – | – |
| #9 | Rebel against authority | 0.86 [0.82; 0.91] | < .001 | 0.80 [0.71; 0.90] | .001 |
| #10 | Consider myself good-looking | 0.87 [0.82; 0.91] | < .001 | – | – |
| #11 | Have a vivid imagination | 0.87 [0.82; 0.91] | < .001 | – | – |
| #12 | Don’t consider myself religious | 1.13 [1.07; 1.19] | < .001 | – | – |
| #13 | Take risks | 0.87 [0.82; 0.92] | < .001 | – | – |
| #14 | Am able to stand up for myself | 0.87 [0.83; 0.92] | < .001 | – | – |
| #15 | Love to think up new ways of doing things | 0.88 [0.83; 0.92] | < .001 | – | – |
| #16 | Am quick to judge others | 1.12 [1.06; 1.18] | < .001 | – | – |
| #17 | Learn quickly | 0.88 [0.84; 0.93] | < .001 | – | – |
| #18 | Am good at many things | 0.89 [0.84; 0.93] | < .001 | – | – |
| #19 | Avoid philosophical discussions | 1.11 [1.06; 1.18] | < .001 | – | – |
| #20 | Consider myself healthy for my age | 0.89 [0.84; 0.93] | < .001 | – | – |
| #21 | Am quick to admit making a mistake | – | – | 1.24 [1.09; 1.40] | .002 |
| #22 | Easily apologize when I have been wrong | – | – | 1.23 [1.09; 1.39] | .002 |
| #23 | Respect authority | – | – | 1.23 [1.09; 1.39] | .002 |
| #24 | Am not embarrassed easily | – | – | 0.79 [0.69; 0.89] | .001 |
| #25 | Am interested in science | – | – | 1.19 [1.06; 1.35] | .007 |
| #26 | Worry about my health | – | – | 1.19 [1.05; 1.34] | .007 |
| #27 | Get upset if objects are not arranged properly | – | – | 0.83 [0.73; 0.94] | .007 |
| #28 | Find it hard to forgive others | – | – | 0.83 [0.74; 0.94] | .006 |
| #29 | Am a submissive person | – | – | 1.16 [1.02; 1.31] | .021 |
| #30 | My interests change quickly | – | – | 0.84 [0.75; 0.95] | .007 |
| #31 | Having good friends is important for me | – | – | 1.16 [1.03; 1.30] | .018 |
| #32 | Prefer to be alone | – | – | 0.85 [0.75; 0.95] | .007 |
| #33 | Have no need for close friendships | – | – | 0.85 [0.75; 0.95] | .007 |
| #34 | Love dangerous situations | – | – | 0.85 [0.75; 0.96] | .009 |
| #35 | Try to provoke others just to get a response | – | – | 0.85 [0.76; 0.95] | .007 |
