# Supplementary material for: Self- and informant-reported personality traits and vaccination against COVID-19
Source: PLoS One. 2024 Mar 14;19(3):e0287413. doi: 10.1371/journal.pone.0287413 (PMC10939290; doi:10.1371/journal.pone.0287413)
Supplement: Supplementary file 13 [file pone.0287413.s013.odt]

S13 Table

NEO-PI-3 Domains and Facets Predicting Vaccination Status

| Personality trait | Source: self | Source: informant |
| --- | --- | --- |
| OR [95% CI] | p | OR [95% CI] | p |
| Neuroticism | 1.10 [0.99; 1.22] | .132 | 1.02 [0.92; 1.13] | .724 |
| Extraversion | 0.97 [0.87; 1.07] | .529 | 1.06 [0.95; 1.18] | .724 |
| Openness | 0.90 [0.80; 1.01] | .132 | 0.98 [0.88; 1.09] | .724 |
| Agreeableness | 1.05 [0.94; 1.16] | .495 | 1.04 [0.94; 1.16] | .724 |
| Conscientiousness | 0.90 [0.81; 1.00] | .132 | 1.04 [0.94; 1.15] | .724 |
| N1: Anxiety | 1.15 [1.04; 1.28] | .090 | 1.08 [0.97; 1.21] | .566 |
| N2: Angry Hostility | 0.98 [0.88; 1.08] | .793 | 0.97 [0.87; 1.08] | .924 |
| N3: Depression | 1.05 [0.95; 1.16] | .609 | 1.08 [0.97; 1.21] | .566 |
| N4: Self-Consciousness | 1.12 [1.01; 1.24] | .136 | 0.95 [0.85; 1.05] | .841 |
| N5: Impulsiveness | 1.09 [0.98; 1.21] | .320 | 1.02 [0.92; 1.14] | .924 |
| N6: Vulnerability | 1.08 [0.97; 1.19] | .391 | 0.99 [0.90; 1.10] | .924 |
| E1: Warmth | 0.96 [0.87; 1.06] | .629 | 1.11 [1.00; 1.23] | .566 |
| E2: Gregariousness | 1.07 [0.96; 1.19] | .493 | 1.05 [0.94; 1.16] | .841 |
| E3: Assertiveness | 0.95 [0.86; 1.06] | .609 | 1.08 [0.98; 1.21] | .566 |
| E4: Activity | 0.96 [0.86; 1.06] | .609 | 0.99 [0.89; 1.10] | .924 |
| E5: Excitement-Seeking | 0.92 [0.81; 1.03] | .391 | 0.98 [0.87; 1.09] | .924 |
| E6: Positive Emotions | 0.98 [0.88; 1.09] | .859 | 1.05 [0.94; 1.17] | .841 |
| O1: Fantasy | 0.94 [0.84; 1.05] | .583 | 0.97 [0.87; 1.08] | .924 |
| O2: Aesthetics | 0.97 [0.87; 1.08] | .769 | 1.01 [0.91; 1.13] | .924 |
| O3: Feelings | 0.95 [0.85; 1.05] | .593 | 1.01 [0.90; 1.12] | .924 |
| O4: Actions | 0.86 [0.77; 0.96] | .090 | 0.97 [0.87; 1.08] | .924 |
| O5: Ideas | 0.88 [0.79; 0.98] | .106 | 0.94 [0.84; 1.05] | .841 |
| O6: Values | 1.03 [0.92; 1.16] | .769 | 1.05 [0.95; 1.17] | .841 |
| A1: Trust | 1.13 [1.02; 1.25] | .106 | 1.09 [0.99; 1.21] | .566 |
| A2: Straightforwardness | 0.99 [0.89; 1.10] | .937 | 1.02 [0.92; 1.14] | .924 |
| A3: Altruism | 1.02 [0.92; 1.13] | .796 | 1.09 [0.99; 1.21] | .566 |
| A4: Compliance | 1.10 [0.99; 1.22] | .263 | 0.99 [0.89; 1.10] | .924 |
| A5: Modesty | 0.95 [0.85; 1.06] | .593 | 1.01 [0.90; 1.12] | .924 |
| A6: Tender-Mindedness | 1.00 [0.89; 1.11] | .937 | 0.98 [0.88; 1.09] | .924 |
| C1: Competence | 0.89 [0.80; 0.98] | .106 | 1.03 [0.92; 1.14] | .924 |
| C2: Order | 0.89 [0.80; 0.98] | .106 | 1.02 [0.92; 1.13] | .924 |
| C3: Dutifulness | 0.99 [0.89; 1.10] | .937 | 1.08 [0.98; 1.20] | .566 |
| C4: Achievement-Striving | 0.87 [0.78; 0.96] | .090 | 0.96 [0.86; 1.06] | .841 |
| C5: Self-Discipline | 0.95 [0.86; 1.05] | .593 | 1.08 [0.97; 1.20] | .566 |
| C6: Deliberation | 0.98 [0.89; 1.09] | .859 | 1.04 [0.94; 1.16] | .841 |
