# Supplementary material for: Self- and informant-reported personality traits and vaccination against COVID-19
Source: PLoS One. 2024 Mar 14;19(3):e0287413. doi: 10.1371/journal.pone.0287413 (PMC10939290; doi:10.1371/journal.pone.0287413)
Supplement: Supplementary file 14 [file pone.0287413.s014.odt]

Trait-level Associations in the Demographic Groups with Lowest Vaccination Rates

	The associations among people without higher education and people in blue-collar occupations were broadly similar to the total sample: Higher odds of vaccination linked to higher self-reported neuroticism and lower self-reported openness. For people without higher education, vaccination status’ associations with items reflecting interest in intellectual activities appeared contradictory: Vaccinated people scored higher on the items “Like to read” and “Find political discussions interesting,” but lower on “Love to think up new ways of doing things” (Table S11). Vaccinated blue-collar workers were rated by informants as more gregarious (higher scores on “Having good friends is important for me,” lower scores on “Prefer to be alone” and “Have no need for close friendships”) and less stubborn (“Easily apologize when I have been wrong,” “Am quick to admit making a mistake;” Table S12). In contrast to vaccinated people without higher education (who “find political discussions interesting”), vaccinated blue-collar workers tended to “avoid philosophical discussions.”
