# Supplementary material for: Self- and informant-reported personality traits and vaccination against COVID-19
Source: PLoS One. 2024 Mar 14;19(3):e0287413. doi: 10.1371/journal.pone.0287413 (PMC10939290; doi:10.1371/journal.pone.0287413)
Supplement: Attachment — Submitted filename: Response to Reviewers.docx [file pone.0287413.s015.docx]

**Revision notes**

We thank the Editor and the Reviewer for directing our attention to the areas of our manuscript that needed improvement. We have revised our manuscript with transparency of the methods and analytic choices, as well as clarity and readability, in mind. We address the feedback in more detail below.

**Editor:** As Reviewer 1 pointed out, some elaboration is necessary in the introduction and discussion sections. On top of that, please provide more explanation for your statement, “Completely separating model training and accuracy testing ensured that predictive accuracies were not overestimated regardless of model complexity.” (rows 297-298). I also wonder why you tested moderation effects by splitting training and testing datasets instead of using more conventional moderation analysis (like interaction effects). Please introduce the “elastic net regression” analysis, as many readers may be unfamiliar with that approach. Why did you choose this method?
**Response:** Following the Reviewer’s suggestions, we have switched the order of the “Vaccination and the personality trait hierarchy” and “Personality traits and vaccination against COVID-19” sections in the introduction, revising the text for logical order of presentation as necessary. We also made various adjustments in the discussion to provide a more compendious summary and to support overall readability. We clarified the statement regarding separation of training and testing data at lines 313-317: by model complexity, we mean the number of predictors; crucially, the more complex model’s accuracy can be overestimated if training and testing data are not properly separated, which we ensured we did. Separating model training and validation is a standard practice in machine learning and other multi-variable approaches (Mõttus et al., 2020, https://doi.org/10.1002/per.2311; Yarkoni & Westfall, 2017, https://doi.org/10.1177/1745691617693393).

We chose the approach to test moderation effects considering our objective to test whether personality traits *together* predict vaccination more strongly between the levels of each moderator (e.g., men versus women). Splitting by data by moderator levels allows testing that (Mõttus et al., 2020, https://doi.org/10.1002/per.2311). In contrast, the more standard approach where the predictors’ (personality domains’ or items’) interactions with the moderator are tested would require a separate interaction effect for each predictor. Not only would this be very computationally intensive (especially in the item models due to the large number of predictors) but it would also not yield an estimate on the moderation effect between the predictors *together* and vaccination. We now clarify the choice of the procedure for testing moderation at lines 357-360 and 363-366 and briefly explain elastic net and its suitability for our objectives at lines 308-313.

**Reviewer #1:** The work presented is serious, relevant and important in the current state of knowledge. It deals with an important and contemporary issue for the prevention of possible future health crises. The results are important and could benefit the entire scientific community.
I cannot comment on the English level of the article, although it is very comprehensible and accessible to non-native English speakers. The English may need to be checked.
There is a problem with the bibliographical references. The references appear as numbers in brackets in the text, but there are no corresponding numbers in the bibliographic list. Also, sometimes the font size changes.

**Response:** We have added the missing numbers to the list of references and ensured that the font size is consistent throughout the manuscript.

**Reviewer #1:** In the introductory section, I wonder whether the part entitled "Vaccination and the personnality trait hierarchy" should not be brought forward to the beginning of the text to better pose the question and make personality a more important moderator (as the results will show later). Otherwise, the introduction is relevant and interesting.

**Response:** We have reconsidered the order of the subsections in the introduction and brought the section on the personality trait hierarchy forward.

**Reviewer #1:** The main reference to the 100NP presentation is missing in the "The Current Study" section, as it is in the "Materials" section. In addition, there should perhaps have been a small review of the literature specific to the 100NP, just as was done on personality concepts.
**Response:** We have added a reference to the 100NP in the Current Study and Materials sections (lines 159 and 236, respectively). We think that given the current study’s objectives it will suffice to briefly mention the studies that have previously used the 100NP, which we have now also added (lines 250-252).

**Reviewer #1:** As for ethical issues, did the participants who completed the questionnaires ten years ago sign a consent form for the data to be used so long after it was collected?
**Response:** The participants gave their written consent to a form that did not specify any restrictions on the time frame during which their answers could be used. This kind of data use is also in agreement with the Estonian Human Genes Research Act (<https://www.riigiteataja.ee/en/eli/508042019001/consolide>).

**Reviewer #1:** In terms of methodology, why were the data of participants with more than 20% genetic links suppressed?

**Response:** Closely related participants were excluded because high relatedness between particpants can inflate the associations; we now note this at lines 200-201.

**Reviewer #1:** What is the boundary between "with higher education" and "without higher education"?
**Response:** As we now also specify in the manuscript (line 299), people were categorized as having higher education if they had obtained at least a bachelor’s or equivalent degree or higher.

**Reviewer #1:** I'm not in a position to accurately assess the statistical analyses presented in this work. Nevertheless, it seems to me that the authors are not pedagogical enough in this section. The article could be improved by accompanying readers a little better in understanding the choice of analyses and results. Even if they seem to be well conducted, it gives the impression of a lack of transparency.

**Response:** We made efforts to make the analyses more accessible: we clarified elastic net to the unfamiliar reader (lines 308-313), clarified the reason for choosing this type of a test of moderation (lines 357-360 and 363-366), and made additional small adjustments throughout the statistical analyses section for intelligibility.

**Reviewer #1:** Please note that in the discussion, having a high score in neuroticism does not mean being neurotic! In fact, researchers now prefer the term "emotional instability" to avoid confusion. Generally speaking, the discussion deserves a summary to get to the point (and use this gain in words to better guide the reader through the results).
**Response:** We have adjusted our wording and replaced “more neurotic” with “less emotionally stable” (line 512). We have added a clearer summary of the study as a whole to the discussion (lines 521-523) and made some additional adjustments for readability.

**Reviewer #1:** Finally, the acknowledgements are not really acknowledgements at all, since they provide access to the study plan.

**Response:** We have removed the links to the study plan and code from the acknowledgements section; they can be found under “Transparency and openness”.
