# Supplementary material for: Self- and informant-reported personality traits and vaccination against COVID-19
Source: PLoS One. 2024 Mar 14;19(3):e0287413. doi: 10.1371/journal.pone.0287413 (PMC10939290; doi:10.1371/journal.pone.0287413)
Supplement: Attachment — Submitted filename: Response to Reviewers.docx [file pone.0287413.s016.docx]

**Revision notes**

We thank the Reviewer for suggesting an additional analysis plan and raising important points regarding the interpretation and contextualization of our results. We have made several adjustments to our manuscript which we describe below.

**Reviewer 2:** The goal of the present paper was to assess whether Covid-19 vaccination predictions are better (more accurate) when using individual/specific (what the authors describe as a diverse set of narrower traits & facets) rather than general/broader (e.g., Big 5) personality characteristics. The researchers also explored the measurement of these characteristics near to the time of one’s vaccination decision versus temporally further away (i.e., 10 years prior) and compared one’s self reports to the reports of a close other. In sum, I think the results are very interesting and that the paper is promising. However, I do have some concerns and suggestions for improvement and list them below.

1. I thought the results were interesting, but did you think about running a hierarchical linear analysis or some form of structural equation modeling, where some factors (predictors) come in early while others come in late? That could potentially boost predictive accuracy and control for potential overlapping variance.

**Response:** Thank you for the idea of adding predictors at different times. We think this approach would benefit from a solid conceptual basis to determine the appropriate order for the predictors and this more nuanced analysis would be better addressed in a separate paper. For example, should later personality traits be added to models with earlier traits or the other way around? What complicates things further is that the samples have very different sizes, and desirably for cross-validation purposes, only partly overlap, lowering the amount of data the models could capitalize on. Overlapping variance among traits is also addressed in elastic net models which select and shrink predictors. So, we gave this idea considerable thought, but we respectfully decided to not model all assessments in the same model.

**Reviewer 2:** 2. I don’t necessarily agree with your comment about liberals following rules more so than conservatives; I think it was overly broad. It could be that both groups follow rules, but it might depend on the context (or one’s ideology). For example, burning down cities and killing other citizens is against the law in most countries, but a couple years ago I saw liberals in the U.S. arguing that it was OK/justified (and that people shouldn’t be put in jail or least not for very long) given it was in protest of systemic racial discrimination. It could also be the case that it depends on the source of the rules/laws. Liberals tend to support rules pushed by the government while conservatives tend to support rules that were established by God.

**Response:** We entirely agree that rule-following could be context-dependent. However, upon reviewing our manuscript, we could not find any instance where we suggest that liberals follow rules more than conservatives. Specifically, in the discussion of liberalism and rule-following, we focus on our results, namely the associations of vaccination with liberalism and rule-following:

- “Vaccinated people were more likely politically liberal (“Support liberal political candidates,” “Don’t consider myself religious,” “Believe that everyone should have a say,” “Treat all ethnicities and religions equally”), yet more conventional (“Like to be viewed as proper and conventional”), less eccentric (“Am considered to be kind of eccentric”), and more respectful of rules and authority (“Respect authority”) compared to unvaccinated people” (results; lines 478–484)
- “In line with previous results (46,49,52,54), vaccinated people tended to be more politically liberal. However, they also had characteristics that, in contrast to liberalism, are commonly associated with lower openness: On average, vaccinated people were more conventional and more respectful of rules and authority. It may make sense that people who respect rules and follow authority would get vaccinated, given that vaccination is generally advised by government and health authorities.” (discussion; lines 603–608)

We see how the pattern of associations – people who were by some indicators more liberal were also more likely to vaccinate – may lead to the impression that liberal people are more rule-abiding because vaccination can be seen as abiding to the rules. However, we refrained from making that leap, not least because other evidence quoted above does not support this. As a result, we are grateful to the Reviewer for raising this point, but there is currently nothing we can change in the manuscript.

**Reviewer 2:** 3. One thing you didn’t spend a lot of time on is trust; obviously some individuals are more trusting of what they see and hear than others. During Covid-19 there was a lot of talk about the “truthfulness” of the info and what constituted misinformation. And public health officials and governments certainly didn’t do themselves any favors in terms of trust when they pushed notions like (my paraphrase) “natural immunity doesn’t work”, “the jab will prevent you from getting covid”, and “people who have gotten the jab won’t transmit covid to others” (or as some claimed “it’s a pandemic of the unvaccinated”), many of which turned out to not be true. But perhaps the vaccinated had more innate trust in others (or institutions) than the unvaccinated.
**Response:** We thank the Reviewer for this comment. We have briefly discussed the role of trust in science in the context of the correlation between vaccination and evidence-based worldview:

- “Most strongly, however, vaccination was related to an evidence-based worldview that may not fit well with the core definitions of any FFM domains. Vaccinated people agreed with statements like “Believe that all events can be explained scientifically” and “Am interested in science.” Similarly, vaccination intention is predicted by trust in science (55), but the phrasing of these science-related items we used implies an association with belief in science in principle (not necessarily in practice). Either way, presenting sound evidence on vaccines’ safety and effectiveness should remove one potential barrier—uncertainty about the risks and benefits of vaccination—among people with an evidence-based worldview, but such messaging might not be as effective for those least likely to vaccinate because these people may not heed or trust the messaging.” (lines 612–621)

However, we note that scores on the Trust facet of the NEO-PI-R did not predict vaccination, so the role of general trust in vaccination behavior (as opposed to the rhetoric around vaccination) remains unclear.

**Reviewer 2:** 4. I also took issue with some of the statements you made about blue collar workers. They may be harder to persuade, but unless you can cite evidence to the contrary, I disagree with your notion that (1) they don’t like to admit when they’re wrong or apologize for their mistakes, and (2) they show little tendency to question or rethink their positions. Perhaps it’s better to say that they need more evidence or maybe stronger evidence to update their beliefs (ie, they’re more staunch in their beliefs/set in their ways), whereas white collar workers (who also tend to be more educated and liberal) tend to change their beliefs more often because they have a lower evidence threshold to exceed. I recently saw some neurological evidence that supports this (see, for example, the work of Christman and Jasper). They argue that inconsistent-handers (who have more cross-talk between the right and left brain hemispheres and tend to be politically more liberal) have larger corpus callosa than consistent-handers (who tend to be more conservative).
**Response:** We should clarify that while vaccinated blue-collars reported more readily admitting and apologizing for their mistakes according to our results, we do not suggest that blue-collar workers do not like to admit being wrong, refuse to apologize for their mistakes, or show little tendency to question or rethink their positions. However, to avoid leaving a wrong impression, we now have replaced our statement about blue-collar workers being hard to persuade with their potentially needing more or stronger evidence. The revised discussion section on blue-collar workers reads:

- “Among those in blue-collar occupations, vaccinated people were more likely to report admitting and apologizing for their mistakes. To the extent that refusing to admit one’s mistakes is an indicator of stubbornness more broadly, compared to white-collar workers, unvaccinated blue-collar workers may need more vaccination-favoring evidence to update their beliefs accordingly, especially when they are also confident and have a low tendency to question or rethink their positions. This may be particularly problematic if blue-collar workers have higher risk of being exposed to the virus—that is, it may not be possible for them to work remotely like it is for many white-collar workers.” (lines 659–668)

**Reviewer 2:** 5. I think the statements you make about application of your findings could be improved. For example, you argue that public health campaigns might be tailored to different sub-groups. It sounds reasonable on the surface, but given that public health campaigns typically target the entire population, how would you pull this off? And if you could pull it off, how would you identify individuals with certain personality characteristics. In another example, you suggest that for the unvaccinated we “normalize” vaccination in the context of spiritual beliefs and practices among religious communities. To some extent, this was tried with Covid-19 (e.g., Billy Graham’s son promoting the vaccine in the U.S.), but it seemed to backfire when people who got the vaccine started dying in droves.
**Response:** We clarify that rather than identifying individuals with certain personality characteristics (which would indeed require additional effort), campaigns could be designed to target the major reasons for non-vaccination, combining knowledge of relevant traits in demographic subgroups with vaccination rates in those subgroups. We made this clearer in the introduction:

- “While some personality trait–vaccination relations may turn out to generalize across individuals’ demographic backgrounds, other traits could be more relevant for vaccination decisions in some groups than others. There is already evidence that personality traits’ associations with vaccination willingness may depend on age and sex. For instance, traits reflecting risk aversion and self-interest could drive older people to vaccinate, while prosocial traits could be more relevant for young people, particularly men (19). If such moderation effects of age, sex, or other demographic variables exist, knowing them could provide further information about the specific drivers of and hindrances to vaccination, potentially allowing for more targeted interventions. For instance, if more anxious older people are apprehensive of vaccines, then campaigns, especially those targeted at this demographic group, could focus on vaccines’ safety or risk-benefit trade-offs; if younger men’s decision to vaccinate is linked to prosociality, campaigns could appeal to altruistic motives, especially if targeted at this demographic group. It would be especially valuable to understand what personality traits are most strongly related to vaccination in groups with the lowest vaccination uptake because effectively motivating these people could have the highest impact on overall vaccination rates. Vaccination campaigns could thus prioritize messages most likely to be effective in subgroups with the lowest vaccine uptake.” (lines 112–127)

**Reviewer 2:** 6. Lastly, do you think your results would generalize to other countries, e.g., the U.S.?

**Response:** We have revised our discussion of generalizability to enhance its clarity. The beginning of our limitations section now reads as follows:

- “Interpretations and applications of the results should be mindful of some potential constraints on generalizability. First, whether the results generalize to other populations is unclear as the associations between specific nuances and vaccination may strongly depend on sociocultural context. Second, because the samples primarily consisted of the ethnic majority (Estonians), results may also not fully generalize to other ethnic groups in Estonia. Third, vaccinated people were somewhat overrepresented in the present study: Vaccination coverage among Estonian adults was 76% as of November 2022 (71% at the beginning of the year), but was 87% and 86% in the 100NP and NEO-PI-3 samples, respectively. This unequal representation suggests that some unvaccinated people were reluctant or otherwise unlikely to become gene donors or participate in the personality studies. Should these people differ in their personality traits from the unvaccinated study participants, this could bias the personality trait–vaccination associations and limit the results’ generalizability to all unvaccinated people. In this case, the bottom line is that the reported associations only reflect *some* people’s personality-based barriers to vaccination.” (lines 694–706)

**Reviewer 2:** 1. I noticed there were some places where the grammar could be improved, and as I recall, there were a couple incomplete sentences. But these are easily fixed.

**Response:** We have reread the manuscript and made adjustments to improve the language.
